# Concordance of coverage estimates from routine and survey data of measles second dose vaccine in Western Kenya

**DOI:** 10.3389/fepid.2025.1663372

**Published:** 2025-09-11

**Authors:** Angela K. Moturi, Moses M. Musau, Samuel K. Muchiri, Peter M. Macharia, Robert W. Snow, Emelda A. Okiro

**Affiliations:** ^1^ Faculty of Public Health and Policy, London School of Hygiene and Tropical Medicine, London, United Kingdom; ^2^ Population and Health Impact Surveillance Group, Kenya Medical Research Institute (KEMRI)-Wellcome Trust Research Programme, Nairobi, Kenya; ^3^ Institute of Life, Earth, and Environment (ILEE), University of Namur, Namur, Belgium; ^4^ Department of Public Health, Institute of Tropical Medicine Antwerp, Antwerp, Belgium; ^5^ Nuffield Department of Medicine, Centre for Tropical Medicine and Global Health, University of Oxford, Oxford, United Kingdom

**Keywords:** demographic and health surveillance, routine health data, geostatistical modelling, child immunisation, measles

## Abstract

**Background:**

Missed opportunities for key vaccinations continue to exacerbate disease outbreaks. Accurately monitoring immunisation coverage is fundamental to identifying gaps in vaccine delivery and informing timely action. This study assesses the agreement between routine and survey-based coverage estimates for the second dose of the measles vaccine (MCV2) in Western Kenya.

**Methods:**

This study utilised model-based geostatistics estimates MCV2 coverage from the 2022 Kenya Demographic and Health Survey (DHS), monthly immunisation data from routine health information systems (2019–2022) imputed for missingness and population data from WorldPop for 2019 across 62 Western Kenyan subnational areas (sub-counties). Routine MCV2 coverage was computed using MCV2 doses as a numerator and two separate denominators: (i) Pentavalent 1 doses to account for children already receiving prior vaccines at health facilities (service-based coverage) and (ii) surviving infants to account for all eligible children (population-based coverage). Concordance was assessed using the 95% confidence intervals (CIs) of survey-modelled estimates, intra-class correlation coefficient (ICC), and Bland-Altman (BA) plots.

**Results:**

Survey-modelled estimates differed substantially in 55 (89%) and 39 (63%) sub-counties compared to population and service-based coverage estimates respectively. The different approaches showed poor congruence in survey-modelled vs. population-based coverage estimates (ICC: 0.10, *p* = 0.229) and survey-modelled vs. service-based coverage estimates (ICC: 0.42, *p* = <0.001); there was moderate congruence of population vs. service-based coverage estimates (ICC: 0.65, *p* = <0.001). Survey-modelled vs. population-based coverage estimates showed the highest bias in BA plots of 18.80 percent points (p.p) compared to 11.02 p.p. and 7.79 p.p. between survey-modelled vs. service-based coverage and population vs. service-based coverage estimates, respectively.

**Conclusions:**

Substantial discrepancies among survey-modelled, routine population, and service-based coverage estimates expose important variations in each approaches’ results. While all approaches offer distinct insights, improving survey models, routine data quality and refining estimates of population catchment is imperative for reliable fine-scale vaccine delivery monitoring.

## 1 Background

Despite improved access to life-saving vaccines, there remains geographic areas of incomplete-vaccination and the perennial problem of zero-dose children who remain under the radar of vaccine programmes (1, 2). These children represent missed vaccination opportunities, resulting in persistent pockets of infection and disease within populations. Resurgences and outbreaks of vaccine-preventable diseases (VPD), such as measles and polio, are on the rise, threatening gains already made towards elimination (3–5).

Measles is a viral respiratory disease spread through contact with the nasal and throat secretions of the infected person through coughing and sneezing. Upon infection, measles can cause serious complications, which can result in death. Vaccines are the main preventive method available to populations, playing a pivotal role in reducing the disease burden. In Kenya, the measles-containing vaccine (MCV) is administered as a measles-rubella combination and is delivered in two doses at 9 (MCV1) and 18 months of age (MCV2). The second dose of the vaccine was first introduced into the Kenya's Expanded Programme of Immunisation (EPI) schedule in 2013 following sustained high coverage of the first dose and has been complemented by periodic Supplemental Immunisation Activities (SIA) targeting cohorts of children under 5 or under 15 (6–8).

Measles remains a significant public health challenge in Kenya, with increasing outbreaks since 2015 (5). Over 1,700 measles cases were recorded in 2022 alone, the highest recorded case burden since 2011 (9). Given the high coverage of other vaccines and geographic access to health services in Kenya (10, 11), it is likely that current measles outbreaks are being driven by fractions of the population that remain under-served or marginalised from routine healthcare services. Since its rollout, MCV delivery has struggled to achieve the recommended target of ≥95% coverage for both doses, which is necessary to achieve elimination (3). In 2021, 22 counties (subnational areas) in Kenya were identified as high-risk areas for measles and targeted in catch-up vaccination campaigns to prevent further outbreaks and fill vaccination gaps (12). Despite this, the 2022 Demographic and Health Survey (DHS) estimated the national coverage of MCV2 at only 67%, with significant variation at the county level (11).

Monitoring immunisation coverage is fundamental to the progressive realisation of universal health coverage (13). There have been improvements to the various available approaches used to estimate health service coverage over time. To track progress over time, immunisation programmes typically rely on estimates of coverage aggregated at the national and regional levels obtained through routine health facility data and/or cross-sectional household surveys. However, surveys occur every 3–5 years, which limits their application in interim years; they are typically powered to be representative at broad subnational level but may not have representative sample sizes of specific sub-groups of the population (14). Further, their use of both recollected vaccine administration and recorded vaccination may introduce recall bias, erroneously increasing estimates of actual coverage. Conversely, routine vaccination data suffers from poor data quality, incomplete reporting and inadequacies in defining denominators obscuring true coverage (15–17). Despite the uncertainties in monitoring, vaccine coverage at broad scales has improved over time. Thus, there is an increasing need for finer-scale estimates that highlight coverage heterogeneities to inform tailoring strategies to reach under-vaccinated children at the local level (4, 18).

To improve fine-scale vaccine coverage predictions, geostatistical techniques are increasingly being utilised to model coverage using survey data (19–24). These interpolation models account for two key components. First, the models incorporate explanatory factors (covariates) such as distance to healthcare facilities, population density, and socioeconomic factors, to predict coverage at high spatial resolutions. Secondly, the models leverage the principle that data at nearby locations are more likely to have similar values compared to more distal locations to explain the variation in coverage that is not accounted for by the selected covariates (25). The resultant gridded surfaces can be aggregated to various administrative levels to inform programme implementation (22, 26).

Considering the various applications of survey-based models and routine data in informing immunisation programme strategy, it is important to assess the level of agreement between these estimates at a subnational scale. This study aims to better understand the consistency of available estimates in quantifying measles second-dose vaccine coverage in Western Kenya.

## 2 Methods

### 2.1 Study context

The present study focused on Western Kenya due to a rising burden of measles in the region and its recent inclusion in MCV vaccination catch-up campaigns (12). Furthermore, it is the only region in Kenya selected to deliver the malaria vaccine (RTS,S/AS01) which includes a 3rd dose at 9 months and a 4th dose at 24 months (27, 28).

Western Kenya comprises 8 administrative counties, namely Bungoma, Busia, Homa Bay, Kakamega, Kisumu, Migori, Siaya, and Vihiga, which are further subdivided into 62 sub-counties (Supplementary Figure S1). Since the promulgation of the 2010 constitution, healthcare service management and delivery have been devolved to county governments. County governments are now responsible for the allocation of resources across sub-counties within their jurisdiction and sub-counties are the reporting units for county health management teams. Granular information at the sub-county level thus provides a more accurate assessment of health trends and disparities and informs resource allocation by county governments.

Vaccinations in Kenya are delivered free of charge at various health facilities per the EPI schedule (29). Health facilities are owned and managed by various providers, including the Ministry of Health (MoH), non-governmental organisations (NGO), faith-based organisations (FBOs), private-for-profit entities and semi-independent institutions such as schools, parastatals, military and prison entities that provide services to select populations. Facilities are classified into 6 levels based on the increasing complexity of service provision, ranging from community health units (Level 1), primary care facilities (Level 2–3), secondary care facilities (Level 4–5) and national referral facilities (Level 6) (30).

### 2.2 Data sources and matching

Kenya's survey-modelled MCV2 coverage estimates were obtained from the publicly available DHS Spatial Data Repository platform (31) and routine MCV2 coverage data was obtained from the Kenyan Health Information System (32). The two data sources provide different levels of granularity and required matching to ensure that the coverage estimates obtained were directly comparable, as described in more detail below.

#### 2.2.1 Kenya demographic and health survey

The Kenya DHS was conducted nationally from February to July 2022 and used a two-stage stratified sampling design to select households nationally across all 47 counties first stratified into urban and rural areas. Clusters were randomly selected from the Kenya Master Household Sample Frame, after which 25 households were surveyed in each cluster. In Western Kenya, 289 clusters were sampled with each sub-county having at least 2 clusters with a maximum of 25 households in each cluster (11).

Data for selected vaccine antigens was recorded for surviving children aged 0–35 months. MCV2 vaccination status was recorded for a sub-group of children aged 24–35 months. This age group is suitable for assessing MCV2 vaccination as children become eligible for the second dose at 18 months of age and are expected to be vaccinated before their third birthday, taking into consideration any possible vaccination delays. The vaccination status of children was confirmed using either a written record, such as the mother and child health handbook, or the mother's verbal report when a written record was unavailable.

For the two most recent household surveys in Kenya (2014 and 2022), the DHS has made publicly available interpolated mapped surfaces (5*5 km resolution) developed from geostatistical models (31). The surfaces are developed utilising data from sampled clusters and Model-Based Geostatistical (MBG) approaches. MBG utilises spatial interpolation techniques to estimate vaccine coverage at unobserved locations based on data points from surveyed children. The DHS approach included covariates such as urbanicity, rainfall, population density, and travel time to cities in the modeling process. Predicted MCV2 vaccination coverage mapped surfaces for Kenya include the estimates of coverage and width of the 95% credible interval. It is essential to note that only 254 out of the 289 clusters sampled in Western Kenya in 2022 had children in the 24–35-month age group (Supplementary Figure S2A). Western Kenya data was extracted from the national surface using the Zonal Statistics tool in ArcGIS 10.8.2 (ESRI Inc., Redlands, CA, USA) (Supplementary Figure S2B). This tool allows the calculation of descriptive statistics such as mean, median, and range values. Specifically, the population weighted mean zonal statistic was used to aggregate the survey-modelled MCV2 vaccination coverage surface at the sub-county level to obtain the average estimate of MCV2 coverage per sub-county. The 2022 population estimates for children aged 24–35 months from WorldPop were used to weight the MCV 2 coverage estimates.

#### 2.2.2 Routine data

The selection of a suitable denominator to evaluate service-based coverage was guided by vaccine coverage estimates from the 2022 DHS survey. Vaccines administered early in the EPI schedule such as BCG and Penta 1 both had near-universal coverage (99%–100%) across Western Kenya (Supplementary Table S1). However, the timing of BCG vaccine administration, immediately after birth, biases its delivery to hospitals with available maternity services whose distribution varies widely across sub-counties. Thus, Penta 1 vaccine data provides a more comprehensive baseline for children active in the vaccination schedule (hereafter referred to as EPI users). As Penta 1 is administered at 6 weeks after birth, it indicates active care-seeking for immunisation services usually at facilities closer to homesteads (28, 33, 34).

Routine immunisation data was extracted from the District Health Information Software (DHIS2), an open-source, web-based platform for reporting all health data in Kenya (35). Facilities report monthly aggregated data of vaccinations administered through routine delivery and campaigns such as SIAs but do not include finer details of the ages of children vaccinated in each month. Immunisation data was thus extracted accounting for when the oldest child (35 months at the start of the survey period-Feb 2022) and youngest child (24 months at the end of the survey period-July 2022) would have likely received their Penta 1 and MCV2 vaccination dose, assuming timely delivery. This approach ensured that the routine data obtained closely matched the surveyed cohort in the KDHS. Facility-level monthly reports of total Penta 1 and MCV2 vaccines administered in Western Kenya were thus obtained across 34 months between April 2019 and January 2022. Each vaccine has 17 months of data corresponding to the expected delivery timeline (Supplementary Figure S3).

This study identified EPI service providers based on submitted reports on DHIS2 of administering any vaccine of interest over the study period. Each facility was further matched to an updated national health facility database (10) to obtain information on ownership, facility level, type and geographic coordinates unavailable from DHIS2. Exclusions were made for facilities such as specialists, facilities newly opened (after January 2022) and those that did not report a single vaccination over the study period (Supplementary Figure S4).

##### Handling of missing data

Routine health information systems in Kenya are susceptible to poor completeness and outliers (33, 36, 37), which may impact estimates obtained from this source. To address this, outliers, defined as values exceeding the median ± 3 times the median absolute deviation (MAD) (38), were identified and replaced with the median value of the health facility. Thereafter, multiple imputation (MI) techniques were used to fill in missing data (39). Specifically, this study employed a multivariate imputation using chained equations (MICE) approach, which leverages existing health facility data and covariates of ownership, type and level of health facility. The model utilised predictive mean matching using the facility's 4 closest non-missing months to determine the closest estimate to fill in missing values.

To limit uncertainty and bias in the multiple imputation (MI) model, only health facilities with complete data for at least 12 out of 17 months (70%) for each vaccine were included in the imputation process based on facility reporting frequency patterns (Supplementary Table S2). This threshold allowed for the imputation of missing data for a majority of vaccinating facilities, thereby maximizing data utilization and ensuring the robustness of the estimates. Facilities falling below this threshold generally reported fewer than 10 doses of either Penta 1 or MCV2 vaccines throughout the study period. Thus, their exclusion from the imputation was deemed unlikely to significantly impact the overall coverage estimates. Given that non-reporting in the DHIS2 platform is indistinguishable from zero reported vaccinations, this pragmatic approach would reduce potential overestimation of vaccines administered. Data imputation was not performed for the months of December 2020 and January 2021. This period coincided with a national health worker strike which, based on data exploration (Supplementary Figure S5), had a greater impact on delivery compared to COVID-19 restrictions, consistent with previous studies (37). The uptick in monthly vaccinations observed in the months following the strike suggest catch-up of children who missed vaccinations during the strike. As such imputation of these months was not carried out as these were true missing values. The imputation model was implemented using the “ICE” package (40) in Stata 17 (41).

### 2.3 Coverage computation

Vaccine coverage is defined as the number of children who receive the MCV2 vaccine relative to eligible populations. This study computed coverage at the second administrative level (sub-counties) using DHIS2 data and two distinct denominators to yield estimates evaluating: (i) the current performance of the health system in retaining children throughout the vaccination schedule (service-based coverage), and (ii) the extent of the immunisation program's outreach within the broader community (population-based coverage).

#### 2.3.1 Service-based Coverage

The denominator comprised the total number of children who received Penta 1 (EPI users) expected to receive MCV2 later in the schedule. Coverage was computed as a fraction with MCV2 recipients as the numerator and EPI users as the denominator and expressed as a percentage.

#### 2.3.2 Population-based Coverage

Annual population estimates of children below 1 year of age are available on the publicly available platform, WorldPop (42). WorldPop combines official census data, satellite imagery such as land cover and dasymetric modeling techniques to produce population datasets that are disaggregated by age at a spatial scale of up to 100 m. Data on children aged <1 year in the year 2019 were downloaded, and population counts were extracted for each of the 62 sub-counties using the Zonal Statistics tool in ArcGIS 10.8.2 (ESRI Inc., Redlands, CA, USA).

##### Population adjustments

Due to the lack of monthly population data for this specific age group, this study assumed a consistent growth rate across the study period and applied an average factor of 1.4 (17/12 months) to the 2019 annual population counts to project the population across the 17 months of administration of MCV2 consistent with the surveyed cohort (Supplementary Figure S3). Further, an infant mortality rate was applied at the county level (11) to obtain an adjusted total of children who survived past the age of 1 and would have received MCV2, as shown below:

$$\begin{array}{rl} \mathrm{Surviving}\;\mathrm{Children} & =\;[\mathrm{Population}\;<1\;\mathrm{yr}\;(2019)\;*\;1.4]\\ & \;*\;(1-\mathrm{IMR}) \end{array}$$

Coverage was computed as a fraction of MCV2 recipients relative to surviving children and expressed as a percentage.

### 2.4 Estimate comparison

To assess the agreement between routine and survey-modelled coverage estimates, a combined approach was used. First, survey estimate confidence intervals computed at the 95% level were used as a benchmark to determine if routine estimates were consistent i.e., when their values were in-between the 95% confidence interval from the modelled survey data. Secondly, congruence between routine and modelled survey-based coverage estimates across sub-counties was explored using the intra-class correlation coefficient (ICC) (43). ICC is a statistic that quantifies the level of agreement between different approaches and takes on values between 0 and 1, where higher agreement is indicated by numbers closer to 1. The ICC was calculated using the single-rater two-way mixed effects model for absolute agreement, with the null hypothesis being ICC = 0 and the alternative hypothesis being ICC > 0. In addition, we adopted the interpretation by Koo & Li (43): if ICC < 0.5, it is indicative of poor agreement; if 0.5 ≤ ICC < 0.75, moderate agreement; if 0.75 ≤ ICC ≤ 0.9, good agreement; and if ICC > 0.9, excellent agreement. Lastly, Bland-Altman (BA) plots with 95% limits of agreement were utilised (44, 45) to examine the concordance of the survey-modelled and routine estimates at the sub-county level. In BA plots, the *x*-axis represents the mean of the estimates across the approaches, and the *y*-axis represents the difference in the estimates, bias (mean of the differences) and limits of agreement (and the corresponding 95% CI calculated as bias ± 1.96 SD, standard deviation). ICC and BA plots were calculated in R software version 4.4.1 (2024-06-14 ucrt) using the packages *psych* and *blandr,* respectively.

## 3 Results

A total of 2,369 health facilities were registered on the DHIS2 platform across all 8 counties of the Western Kenya region. Facilities categorised as specialist (113, 5%), those opened after January 2022 (132, 6%) and those that did not report administering any vaccines over the study period (2019–2022) (626, 26%) were excluded from further analysis as they do not routinely provide immunisation services (Supplementary Figure S4). The remaining 1,498 facilities were confirmed to provide immunisation services and majorly comprised of Ministry of Health facilities (74%) and primary level facilities such as dispensaries and clinics (62%) as summarised in Supplementary Table S3. Overall, facilities demonstrate high consistency in reporting, with 1,258 (84%) and 1,142 (76%) reporting Penta 1 and MCV2 administration for at least 12 months, respectively. However, differences in reporting across antigens are evident as 62% of facilities submitted Penta 1 reports across all 17 months of interest, while only 15% reported MCV2 administration at the same frequency (Supplementary Table S2).

A total of 404,288 doses of Penta 1 and 212,715 doses of MCV2 vaccines were administered during the study period relative to an estimated eligible population of 463,526 children. An average of 22,000 Penta 1 vaccines were administered monthly between April 2019 and August 2020. MCV2 vaccines had a notably lower monthly average of 15,000 between September 2020 and January 2021. Sharp declines in MCV2 delivery were experienced from December 2020 to January 2021, coinciding with the national health worker strike (Supplementary Figure S5). This period was followed by an increase in vaccinations administered, signalling catch-up. MCV2 delivery peaks in June 2021, coinciding with SIA activities across Western Kenya.

**Figure 1:**
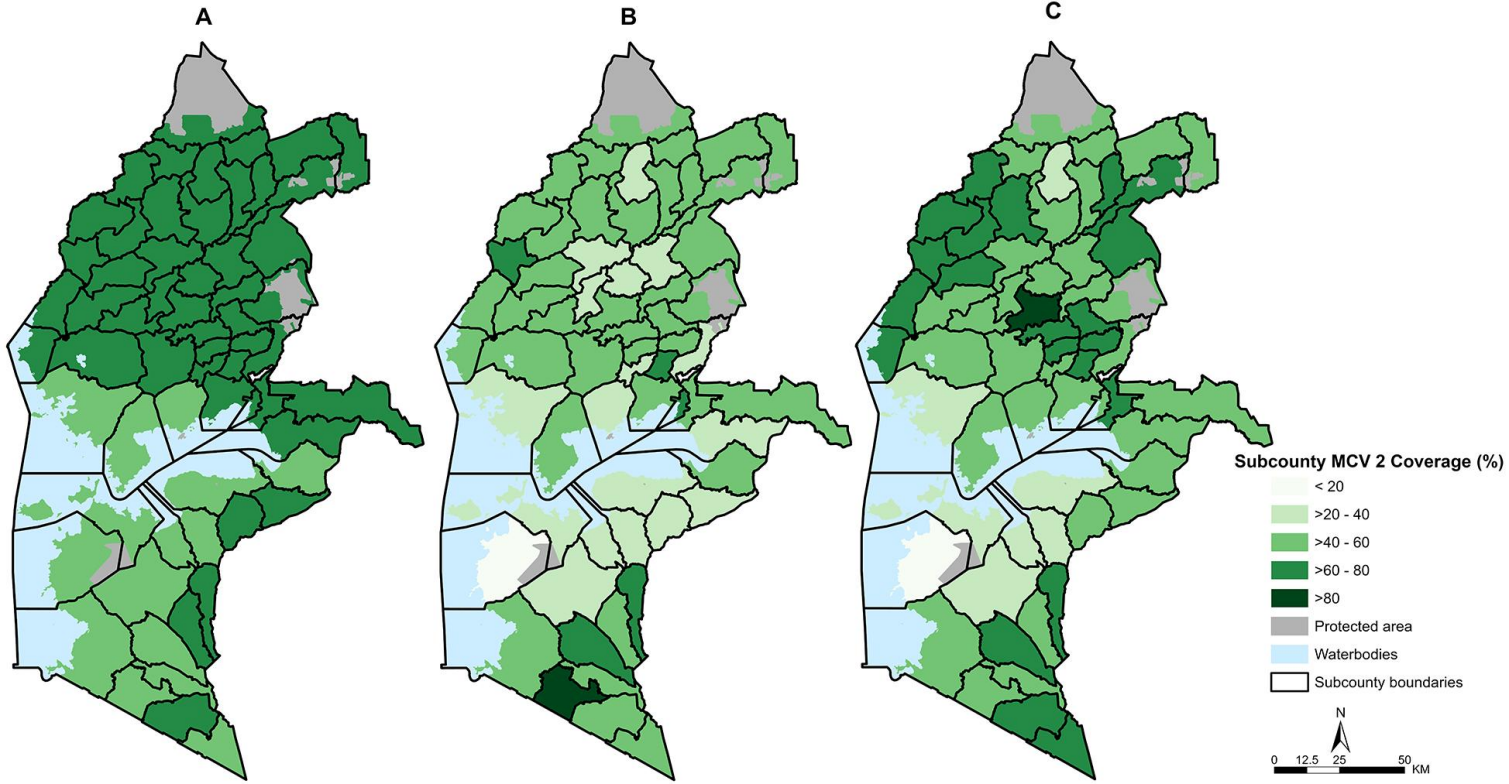
Maps showing sub-county level MCV 2 coverage estimates from survey-modelled **(A)**, population-based **(B)** and service-based approaches **(C)** across Western Kenya.

Overall, all approaches exhibit varying ranges of coverage. Survey-modelled coverage estimates range between 50% and 73% (Figure 1A), population-based coverage is between 18% and 80% (Figure 1B), and service-based coverage varies between 19% and 81% (Figure 1C). Survey-modelled estimates exhibit little variation in coverage across sub-counties, with 47 of 62 sub-counties having coverage of over 60% compared to 22 counties for service-based coverage and only six for population-based coverage. There are similarities across all approaches in the sub-counties ranked with the lowest coverage such as in the southern region of Western Kenya, which broadly covers Homa Bay county (Figure 1). Conversely, the sub-counties with the highest coverage vary across all methods. This is particularly evident in the northern region, whereby the distribution of coverage differs between approaches (Figure 1).

**Figure 2:**
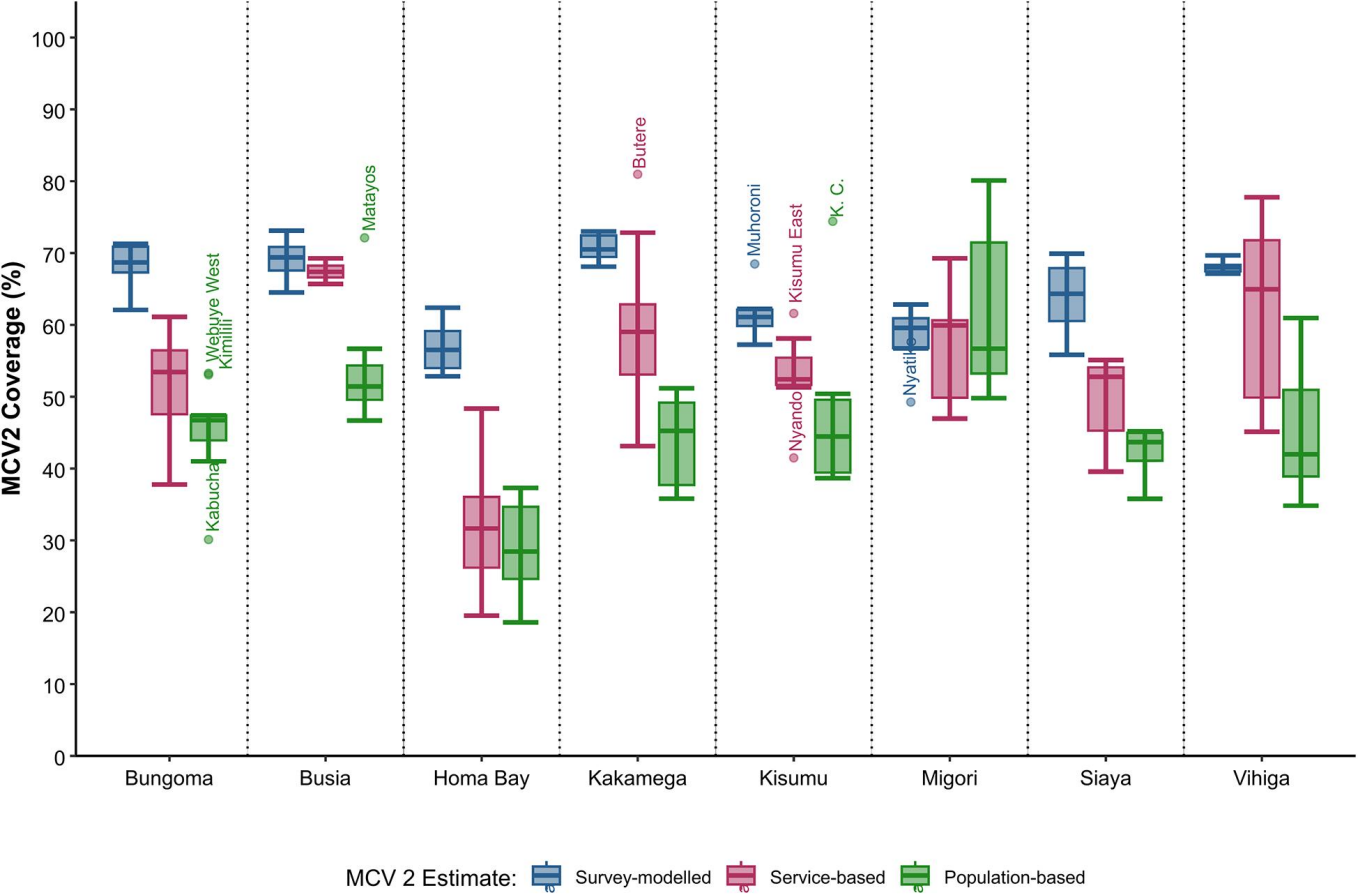
Boxplot showing the range of sub-county MCV 2 coverage estimates (%) across Western Kenya for survey-modelled, service and population-based approaches.

Across sub-counties, survey-modelled estimates typically rank the highest, followed by service-based coverage, with population-based coverage ranking the lowest (Figure 2). Service-based coverage is highest in sub-counties within Busia County, ranging between 66% and 69%, while survey-modelled estimates are highest in Kakamega County at 67%–73%. All approaches indicate that sub-counties in Homa Bay County have the lowest MCV2 coverage. Population-based coverage estimates had the highest number of outliers with five sub-counties identified, namely Webuye West, Kimilili, Kabuchai, Kisumu Central and Matayos (Figure 2).

The differences between the estimates were assessed against the 95% credible intervals of the survey model. The credible intervals, varying from ±11 to ±23 percentage points, reflect the considerable disparity in model performance across the region. Substantial differences were observed between population-based coverage and survey-modelled estimates in 55 sub-counties (89%) and between service-based coverage estimates and survey-modelled estimates in 39 sub-counties (63%) (Table 1). Similar findings are observed in bivariate plots as shown in (Supplementary Figure S6).

**Table 1:** MCV2 coverage estimates for 62 sub-counties in Western Kenya computed using survey-model and routine (population and service denominators) approaches.

County	Subcounty	MCV2 Coverage (%)		
		Survey-model (95% CI)	Population-based (World Pop)	Service-based (Penta 1)
Bungoma	Bumula	71 (64–78)	47**	61**
	Kabuchai	69 (62–76)	30**	38**
	Kanduyi	71 (65–77)	44**	48**
	Kimilili	69 (62–76)	53**	56**
	Mt. Elgon	62 (51–73)	47**	47**
	Sirisia	67 (58–76)	47**	53**
	Tongaren	67 (59–75)	47**	55**
	Webuye East	69 (63–75)	41**	60**
	Webuye West	71 (65–77)	53**	48**
Busia	Bunyala	64 (56–72)	57	69
	Butula	71 (65–77)	48**	66
	Matayos	71 (64–78)	72	68
	Nambale	73 (66–80)	52**	67
	Samia	66 (58–74)	51**	69
	Teso North	69 (62–76)	51**	67
	Teso South	69 (63–75)	47**	66
Homa Bay	Homa Bay	59 (50–68)	37**	30**
	Ndhiwa	56 (46–66)	26**	27**
	Rachuonyo East	62 (55–69)	37**	48**
	Rachuonyo North	54 (45–63)	21**	25**
	Rachuonyo South	61 (53–69)	34**	40**
	Rangwe	57 (48–66)	31**	33**
	Suba North	53 (41–65)	26**	35**
	Suba South	53 (42–64)	19**	20**
Kakamega	Butere	72 (66–78)	51**	81**
	Ikolomani	70 (64–76)	49**	73
	Khwisero	70 (63–77)	44**	66
	Likuyani	68 (61–75)	48**	59**
	Lugari	69 (62–76)	46**	60**
	Lurambi	72 (65–79)	51**	54**
	Malava	69 (61–77)	51**	62
	Matungu	73 (67–79)	38**	48**
	Mumias East	73 (67–79)	37**	58**
	Mumias West	73 (67–79)	36**	49**
	Navakholo	71 (64–78)	36**	43**
	Shinyalu	69 (63–75)	43**	59**
Kisumu	Kisumu Central	60 (53–67)	74**	52**
	Kisumu East	62 (55–69)	50**	62
	Kisumu West	62 (54–70)	49**	53**
	Muhoroni	68 (61–75)	40**	58**
	Nyakach	59 (52–66)	44**	52
	Nyando	61 (53–69)	39**	41**
	Seme	57 (49–65)	39**	51
Migori	Awendo	63 (56–70)	54**	50**
	Kuria East	59 (50–68)	59	61
	Kuria West	60 (52–68)	55	61
	Nyatike	49 (37–61)	50	47
	Rongo	63 (56–70)	76**	69
	Suna East	60 (52–68)	52	50**
	Suna West	57 (48–66)	80**	60
	Uriri	57 (49–65)	70**	60
Siaya	Alego-usonga	63 (55–71)	41**	43**
	Bondo	59 (48–70)	36**	40**
	Gem	65 (59–71)	45**	54**
	Rarieda	56 (47–65)	45**	53
	Ugenya	70 (64–76)	43**	53**
	Ugunja	69 (63–75)	45**	55**
Vihiga	Emuhaya	68 (61–75)	42**	72
	Hamisi	67 (60–74)	39**	45**
	Luanda	68 (61–75)	35**	50**
	Sabatia	70 (63–77)	51**	78**
	Vihiga	67 (60–74)	61	65

**p < 0.05.

Congruence between survey-modelled and routine population-based estimates for MCV2 vaccine coverage across sub-counties based on ICC was 0.10 (95% CI: 0.00–0.34, *p* = 0.229) (Table 2), indicating poor agreement between the two approaches. However, based on the *p*-value and 95% CI, we fail to reject the null hypothesis, ICC = 0; there is no agreement between the two approaches. The ICC estimate for congruence between survey-modelled and routine service-based estimates for MCV2 vaccine coverage across sub-counties is 0.42 (*p* = <0.001) (Table 2), indicating poor agreement between the two approaches; the agreement ranges from poor to moderate (95% CI: 0.20–0.61). For congruence between routine service and routine population-based estimates for MCV2 vaccine coverage across sub-counties, the ICC is 0.65 (*p* = <0.001) (Table 2), indicating moderate agreement between the two approaches; the agreement ranges from (marginally) poor to moderate (95% CI: 0.49–0.78).

**Table 2:** Agreement between routine population and service denominators and survey-modelled estimates of MCV2 vaccine coverage across sub-counties.

Comparison	ICC (95% CI)	*p*-value
Survey-modelled vs. routine population-based coverage	0.1 (0.00–0.34)	0.229
Survey-modelled vs. routine service-based coverage	0.42 (0.20–0.61)	**<0.001**
Routine service-based vs. routine population-based coverage	0.65 (0.49–0.78)	**<0.001**

The bold *p*-values show statistically significant congruence.

**Figure 3:**
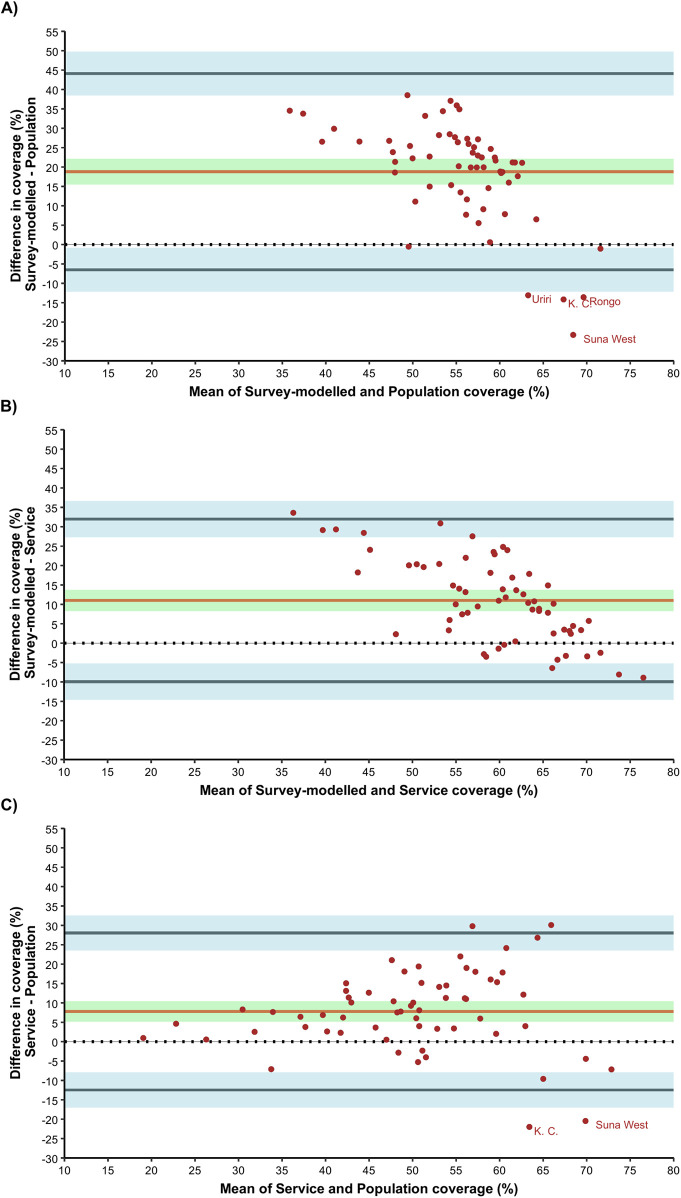
Bland-Altman for agreement analysis; **(A)** survey-modelled vs. population, **(B)** survey-modelled vs. service and **(C)** service vs population.

Similar results were reflected in the BA plot (Figure 3). The estimated bias in MCV2 vaccine coverage estimates across sub-counties between survey-modelled and routine population-based approaches was highest at 18.80 (95% CI: 15.52–22.08) percentage points (p.p) (Figure 3A). This positive bias indicates overestimation in MCV2 vaccine coverage estimates by the survey-modelled approach in comparison to routine population-based approach. In addition, these two approaches had the widest BA limits of agreement ranging from −6.51 to 44.12 p.p. The 95% CI for the lower limit of agreement and upper limit of agreement were −12.14 to −0.87 and 38.48 to 49.75 respectively (light blue area in Figure 3A). Notably, estimates for four sub counties: Uriri, Kisumu Central (K. C), Rongo and Suna West fall outside the lower bounds of BA limits of agreements.

Agreement in MCV2 vaccine coverage estimates across sub-counties between survey-modelled and routine service-based coverage approaches had a bias of 11.02 p.p. (95% CI: 8.30–13.73). Like the comparison with routine population-based coverage, the observed positive bias indicates overestimation in survey-modelled estimates in comparison to routine service-based coverage estimates across the sub counties. The BA limits of agreement for survey-modelled vs. routine service-based coverage ranged from −9.94 to 31.97 p.p. The 95% CI for the lower limit of agreement and upper limit of agreement were −14.60 to −5.27 and 27.30 to 36.63 respectively (light blue area in Figure 3B).

For agreement between routine service and routine population-based estimates for MCV2 vaccine coverage across sub counties, the bias was 7.79 p.p. (95% CI: 5.16–10.41) indicating overestimation in service-based coverage estimates in comparison to population-based coverage estimates across the sub counties. The BA limits of agreement ranged from −12.47 to 28.04 p.p. The 95% CI for the lower limit of agreement and upper limit of agreement were −16.98 to −7.96 and 23.53 to 32.55 respectively (light blue area in Figure 3C). Notably, estimates for Kisumu Central and Suna West sub counties fall outside the bounds of BA limits of agreements.

Further evaluation of the patterns of coverage at the county level reveal persistent disparities in the approaches even at broader scales. The regional average for Western Kenya in the survey-based model estimates is 65%, 54% for service-based coverage and 46% for population-based coverage (Supplementary Figure S7). Also, when compared with the county-level direct survey-weighted estimates from the survey, 7 out of 8 survey-modelled estimates fell within the 95% confidence interval, whereas only 2 out of 8 and 4 out of 8 estimates from the population-based and service-based approaches, respectively, did so (Supplementary Table S4).

## 4 Discussion

Multiple approaches can be used to estimate vaccine coverage using either survey data or routine data, this study has analysed how different estimations of MCV2 coverage compare across 62 sub-counties in Western Kenya. The analysis revealed notable discrepancies between service, population, and survey-based modelled coverage estimates, highlighting the importance of critically evaluating the strengths and limitations inherent to each approach when shaping immunization program strategies and quantifying missed vaccination opportunities. Further, the MCV2 vaccination rates were consistently below 80% among eligible children, including those already engaged in routine EPI services, highlighting significant systemic challenges in routine immunization delivery. Such obstacles impede the attainment of the >95% coverage required for measles elimination, as envisioned in the Immunization Agenda 2030 targets (46).

Population-based estimates consistently yielded the lowest coverage, indicating a higher number of unvaccinated children within the community (Figures 1, 2). However, healthcare-seeking behaviors often extend beyond sub-county boundaries, resulting in increased community coverage through cross-border vaccinations. Previous studies have noted that while some subnational regions may exhibit very low coverage, areas with larger health facilities often display the opposite extreme, with values exceeding 100% due to patient selection and mobility (28, 33, 34). These outliers are typically reconciled when coverage is computed at broader scales, such as the county level. Nonetheless, population-based coverage estimates remain a valuable indicator of sub-counties with persistent low coverage, as shown here for Homa Bay County (Table 1), which would benefit most from intensified routine immunization delivery and targeted campaigns. The increasing availability of gridded population estimates at finer resolutions, such as 1 × 1 km grids reflecting actual population settlement patterns (42), further facilitates the identification of absolute numbers of missed opportunities and the effective targeting of outreach activities. The incorporation of precise residential addresses into routine vaccination records, would be an additional benefit to a more rigorous definition of catchment areas extending beyond administrative boundaries (47, 48), and would substantially refine our understanding of care-seeking behaviors to inform use of appropriate population denominators at finer scales.

Survey-modelled estimates consistently yielded the highest coverage, likely due to their broader inclusion of vaccinations administered outside routine immunization systems, such as through SIAs. Although model-based geostatistical methods aim to address key data gaps by leveraging sparse survey data, the current models for MCV2 coverage estimation exhibit several limitations. Small sample sizes for sub-groups such as 24–35-month-old children will impact on model performance (18, 49), models may not account for survey design weights (50); and the focus on environmental covariates excludes other key parameters able to predict MCV2 coverage including education and poverty (51). Additionally, these prediction estimates are static, applying only to the survey year, thus limiting their ability to inform prompt filling in of vaccination gaps in the manner that routine data would. The construction of these modelled predictions of vaccine coverage must be better understood to interpret their inherent weaknesses and ultimate use in decision-making.

Service-based coverage estimates occupied an intermediate position between population-based and survey-modelled estimates and provided a more specific evaluation of EPI program retention across the immunization schedule which other methods do not account for. As these estimates rely on routine data, they offer a cost-effective approach for health monitoring over time and facilitate timely identification of missed opportunities. A focused effort to target children already engaged in the healthcare system but subsequently dropping out represents a promising strategy for increasing community coverage. Service-based estimates are indispensable for monitoring immunization performance at the facility level, enabling accurate forecasting of vaccine supply requirements and identification of service delivery gaps. However, this approach does not account for hard-to-reach populations not engaged in routine care or those reached via community-based vaccinations and, therefore, should not be viewed as a substitute for community-wide coverage assessments.

Service- and population-based estimates exhibited the highest level of agreement, underscoring their potential for capturing similar target populations (Figure 3; Table 2). This underscores their combined utility in identifying missed opportunities for MCV2 vaccinations at greater frequency. By complementing each other, these approaches mitigate the limitations present in each method. Notably, survey-modelled estimates displayed the least agreement with both service- and population-based methods, consistently yielding higher and more homogenous estimates across the Western Kenya region (Figure 1). This finding aligns with prior studies that have documented discrepancies between routine coverage data and broader community surveys (34, 52), attributable to survey limitations such as the under-sampling of populations in urban slums and remote settlements, who are more likely to be under-immunized (14, 53, 54).

The presence of sub-county outliers across population- and survey-model methods underscores the critical need for cross-validation to identify inconsistencies requiring further investigation. For instance, in Kisumu Central sub-county (Table 1), contrary to the overall pattern, service-based coverage was lower than both population-based and survey-modelled coverage. This observed discrepancy may result from health facilities within the sub-county attracting children from neighboring areas for immunization services, thereby increasing population-based coverage figures relative to the local population initially engaged in EPI services. Unlike service-based coverage, whose denominator reflects vaccinations administered specifically to the population accessing routine services within the sub-county, population-based estimates may include the total of vaccinated children residing in or visiting the sub-county.

The reliance on a single approach for estimating vaccine coverage and identifying missed opportunities has profound implications for decision-making, particularly at the sub-county level. While household surveys are widely regarded as the “gold standard” for coverage estimation, the survey-modeled estimates analyzed in this study demonstrated the least agreement with routine data-based approaches and consistently yielded higher coverage estimates (Table 2; Figure 1) which is consistent with previous work comparing survey and routine approaches (34). Notably, substantial discrepancies in estimates were observed in over 60% of sub-counties, further underscoring the variations in each method. For example, in Suba South sub-county, modeled coverage was estimated at 58%, whereas both service-based and routine-based estimates fell below 21%, representing a striking difference of nearly 40 percentage points (Table 1). Furthermore, in comparison with the county level direct survey-weighted estimates obtained from the survey, only one out of eight county estimates from the survey-modelled approach did not fall within the 95% CI compared to six and four for population-based and service-based approaches respectively. While this comparison strengthens the case for the reliability of the survey-modelled approach relative to routine data, as would be expected, it also highlights that routine data estimates can vary widely from survey estimates. This discrepancy should be acknowledged and taken into account when interpreting coverage data estimates from the individual approaches when making policy decisions as, depending on the approach used, critical immunization gaps within communities may remain obscured and grossly underestimated, perpetuating conditions that allow outbreaks to persist.

Although the methodologies employed in this study differ substantially, they may each capture unique aspects of vaccine coverage. As a result, their combined use provides a more comprehensive understanding of immunization gaps and challenges. Combining these methods can help bridge disparities and inform targeted interventions for improved coverage. For example, using datasets from the different approaches to triangulate vaccine coverage estimates can be done as shown in the case of malaria burden modelling (55). Additionally, evidence from other studies in Kenya offers critical insights into the social and demographic determinants influencing measles vaccine uptake, such as a child's immunization history, household income, and caregiver education level (56, 57). These factors must be carefully considered when devising strategies for delivering vaccines to children beyond one year. Further, systemic and emerging challenges such as healthcare worker shortages, strikes (58), vaccine stock-outs (59), delays in outreach activities (SIAs) due to limited funding and COVID-19 (8) as well as infrastructural limitations, such as inadequate road networks restricting community access to health services, must also be addressed to prevent further missed opportunities. The findings of this study provide decision-makers with evidence to evaluate various estimation methods, enabling the development of improved strategies, informed budgetary allocations, and strengthened routine immunization programs.

Robust data reporting and surveillance systems are essential for identifying and addressing gaps in MCV2 immunization coverage. Enhancing the quality of routine data and incorporating granular data on dynamic population changes, such as migration and varying growth rates, would improve the accuracy of both numerators and denominators used in routine coverage monitoring (23, 60). The observed variability in reporting completeness across vaccination metrics in this study stresses the pressing need for ongoing efforts to enhance the comprehensiveness and reliability of health facility and census data. These improvements are fundamental to fostering an environment where data-driven decision-making informs local administrative and national policy for routine and outreach vaccination programs.

This study has some limitations that must be considered when interpreting the findings. It used data from DHIS2, which has issues with missing information. To address this, multiple imputation was performed to create a complete dataset among facilities that consistently reported their data. Although this does not replace complete data, it does help ensure that the estimates of vaccine coverage more accurately reflect reality, with minimal bias in the imputed values. Additionally, the study relied on gridded population data derived from national census figures. While this source enhances the population measures at smaller scales, its reliability hinges on the accuracy of the original census data. Improving systems like civil registration could help address this data gap. Lastly, the accuracy of the 95% CI from the survey-modelled estimates remains undetermined as the DHS does not report the CIs from cross-validation exercise.

## 5 Conclusion

This study underscores the importance of using various datasets to gain a comprehensive understanding of MCV2 immunisation coverage from multiple perspectives. While discrepancies exist between routine and survey-modelled methods, both approaches yield valuable insights and unique strengths for different aspects of vaccine delivery monitoring, catering to the diverse needs of stakeholders. To enhance the accuracy of vaccine coverage estimations at more granular levels, it is essential to improve the quality of routine data to refine our understanding of service utilization and population dynamics. This would enable accurate identification of underserved populations, thus informing targeted interventions to bridge gaps in vaccine coverage. By addressing the noted shortcomings and geographical inequalities, immunisation programs can achieve broader coverage and better health outcomes for children in the region.

## Data Availability

The original contributions presented in the study are included in the article/Supplementary Material, further inquiries can be directed to the corresponding author.
