# Supplementary material for: Concordance of coverage estimates from routine and survey data of measles second dose vaccine in Western Kenya
Source: Front Epidemiol. 2025 Sep 11;5:1663372. doi: 10.3389/fepid.2025.1663372 (PMC12460269; doi:10.3389/fepid.2025.1663372)
Supplement: Supplementary file 1 [file Datasheet1.pdf]

Supplementary Material

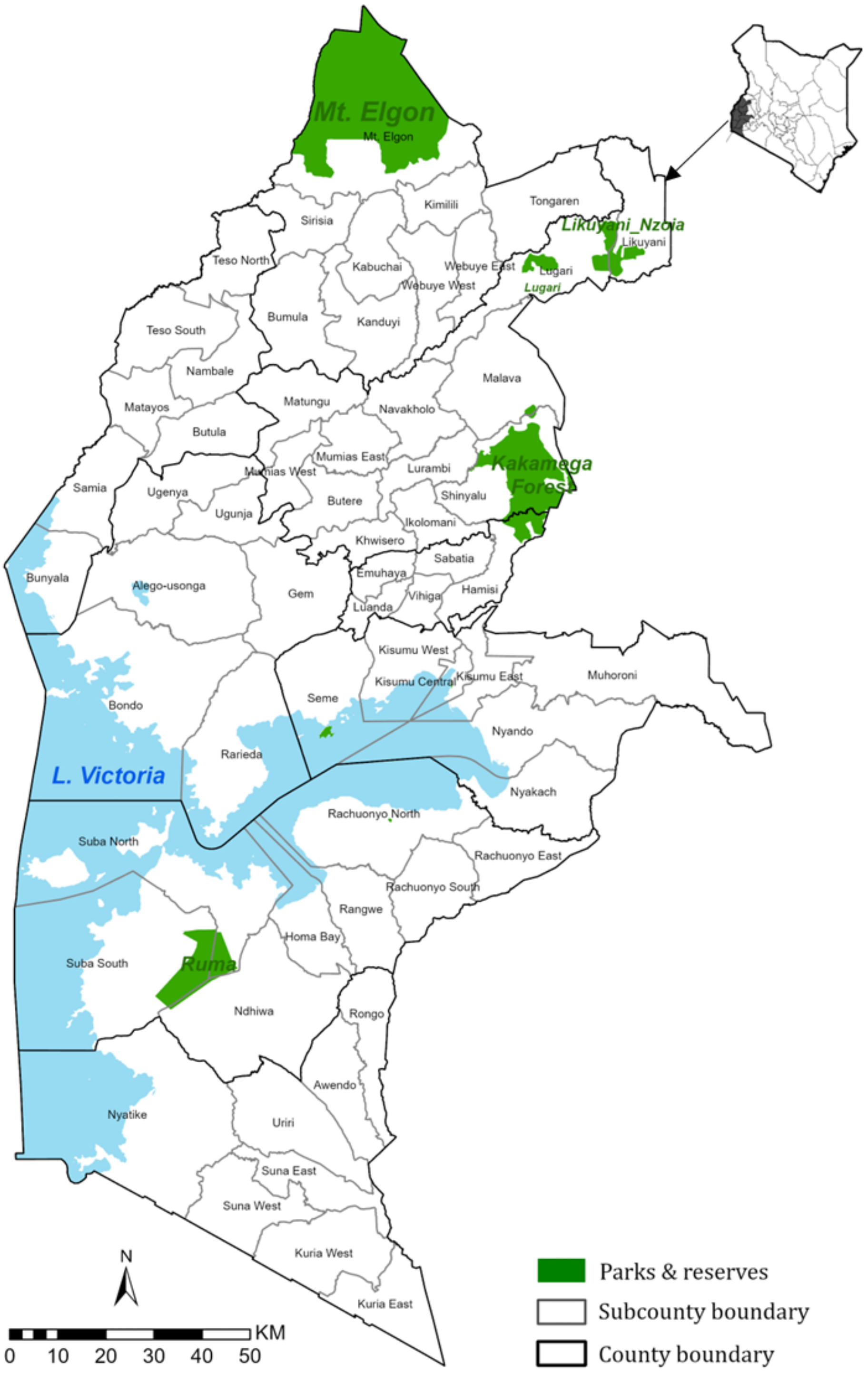

Figure S1: Western Kenya county boundaries

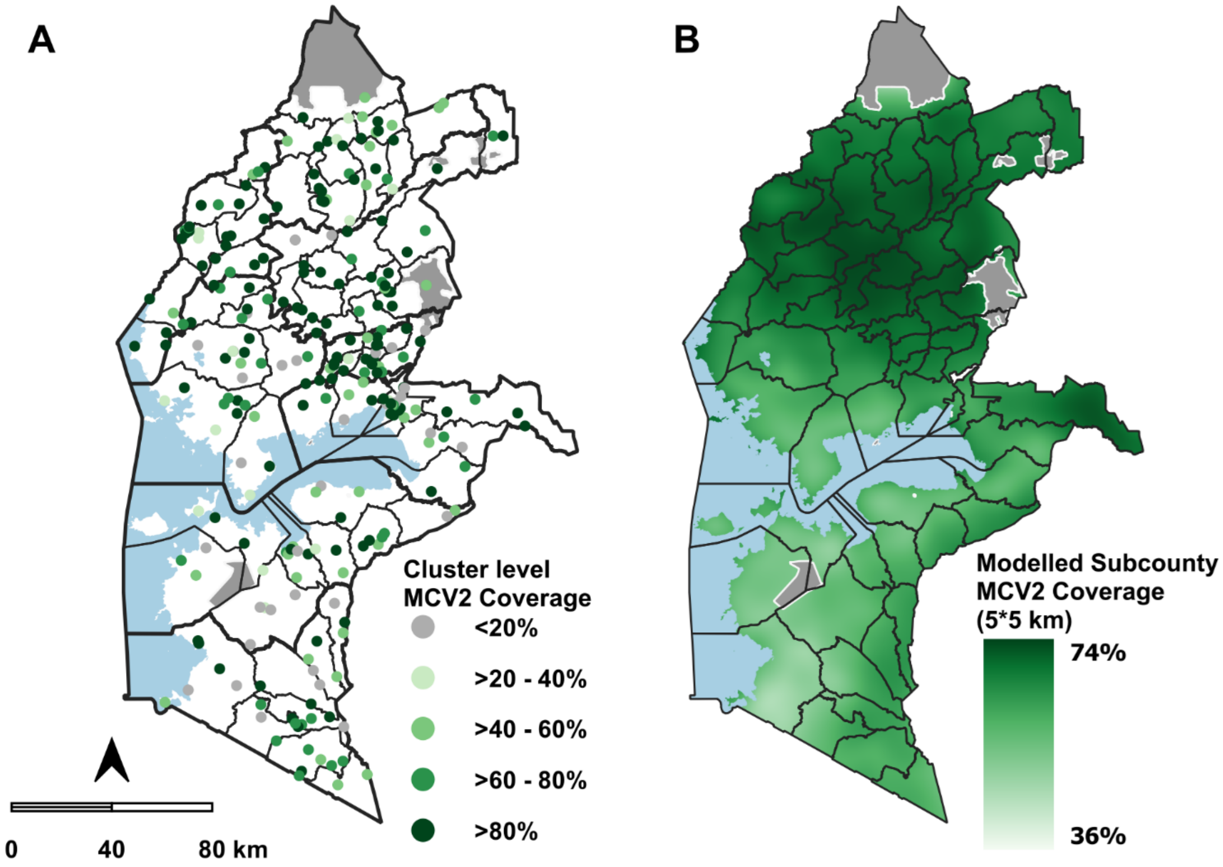

Figure S2: Maps showing the DHS survey estimates of MCV2 coverage at the cluster level (A) and modelled estimates at 5×5 km resolution (B)

Table S1: 2022 Kenya Demographic and Health Survey vaccine coverage estimates for select vaccines across 8 Western Kenya counties

County	BCG (Birth)*	Penta 1 (6 weeks)*	MCV1 (9 months)*	MCV2 (18 months)*
Kakamega	100	100	96	81.6
Vihiga	100	100	100	74.8
Bungoma	100	98.7	91.3	66.9
Busia	98.2	100	90.5	84.3
Siaya	99.3	99.3	94.1	54.1
Kisumu	97.7	100	93.5	64.7
Homa Bay	98.3	97.5	89	46.6
Migori	99.6	99.6	93.1	62.3
National	97	97	89	67

*Time of administration of each vaccine antigen. Source: (KNBS & ICF 2023)

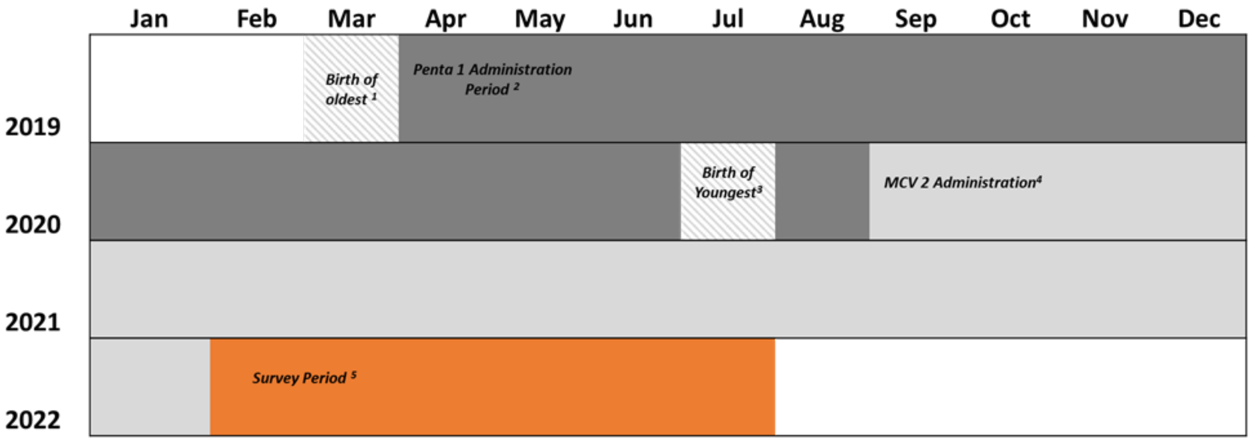

Figure S3: Timelines of data extraction of Penta 1 and MCV 2 vaccine from DHIS2 accounting for age eligibility of the 24–35-month-old children surveyed in the KDHS survey (2022)

*Example: A child born in March 2019 should have received Penta 1 in April 2019 and MCV2 in September 2020, and their cohort, along with other children in that cohort, was surveyed between February and July 2022.

[1]Month in which the oldest eligible child (35 months at the start of the survey) would have been born.

[2]Reference period in which a child aged between 24-35 months during the survey would have been expected to receive the Penta 1 vaccine (April 2019- August 2020).

[3]Month in which the youngest eligible child (24 months at the end of the survey) would have been born.

[4]Reference period in which a child aged between 24-35 months during the survey would have been expected to receive the MCV2 vaccine (September 2020- January 2022).

[5]DHS Survey period in Kenya during which children aged between 24-35 months were sampled and MCV2 vaccination status determined.

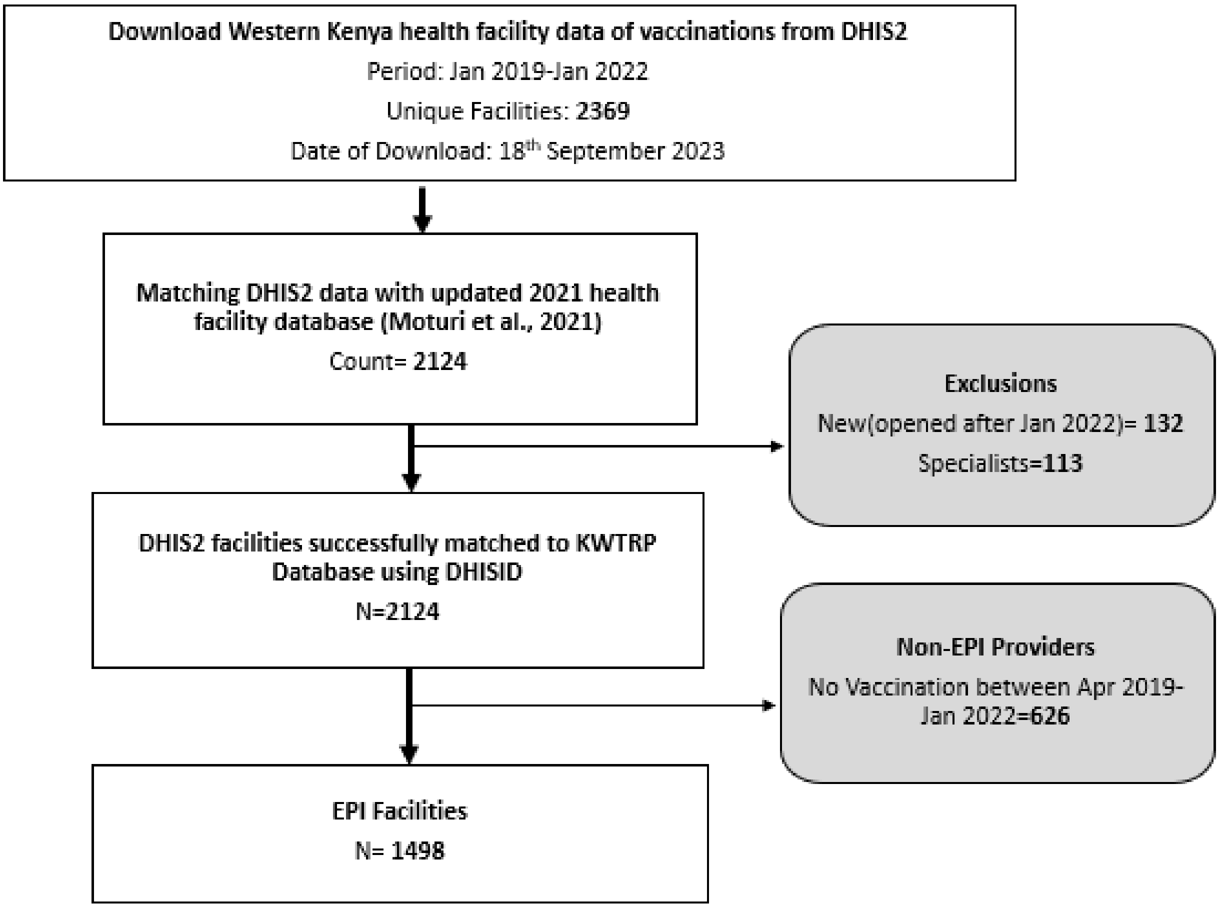

Figure S4: Flowchart illustrating the selection process for identifying vaccinating (EPI) facilities based on routine data reported by facilities.

[1]Specialists include providers that only offer specific medical services such as dentists, eye clinics or family planning and do not routinely administer vaccinations.

[2]Non-EPI facilities comprise health providers that made no report of administering either Penta 1 or MCV 2 antigen on the DHIS2 platform over the entire study period.

Table S2: Distribution of reporting frequencies of EPI service providers in Western Kenya

Vaccine Reporting Frequencies	N (%)
Total Facilities	1,498(100%)
Penta 1 (Apr 19-Aug 20)	
<5 months	180 (12.0%)
5-11 months	60 (4.0%)
12-16 months	324 (21.6%)
17 months	934 (62.3%)
MCV2 (Sep 20- Jan 22)	
<5 months	136 (9.1%)
5-11 months	220 (14.7%)
12-16 months	919 (61.3%)
17 months	223 (14.9%)

Table S3: Characteristics of EPI service providers in Western Kenya

Facility Characteristics	N (%)
N	1,498
Ownership	
MOH	1,105 (73.8%)
Private	209 (14.0%)
Faith Based Organisations	148 (9.9%)
Non-Governmental Organisations	23 (1.5%)
Other (schools, parastatals)	13 (0.9%)
Facility Level	
Level 2	956 (63.8%)
Level 3	384 (25.6%)
Level 4	156 (10.4%)
Level 5	2 (0.1%)
Type	
Clinic	85 (5.7%)
Dispensary	842 (56.2%)
Health Centre	328 (21.9%)
Hospital	158 (10.5%)
Maternity & Nursing Home	40 (2.7%)
Medical Centre	45 (3.0%)

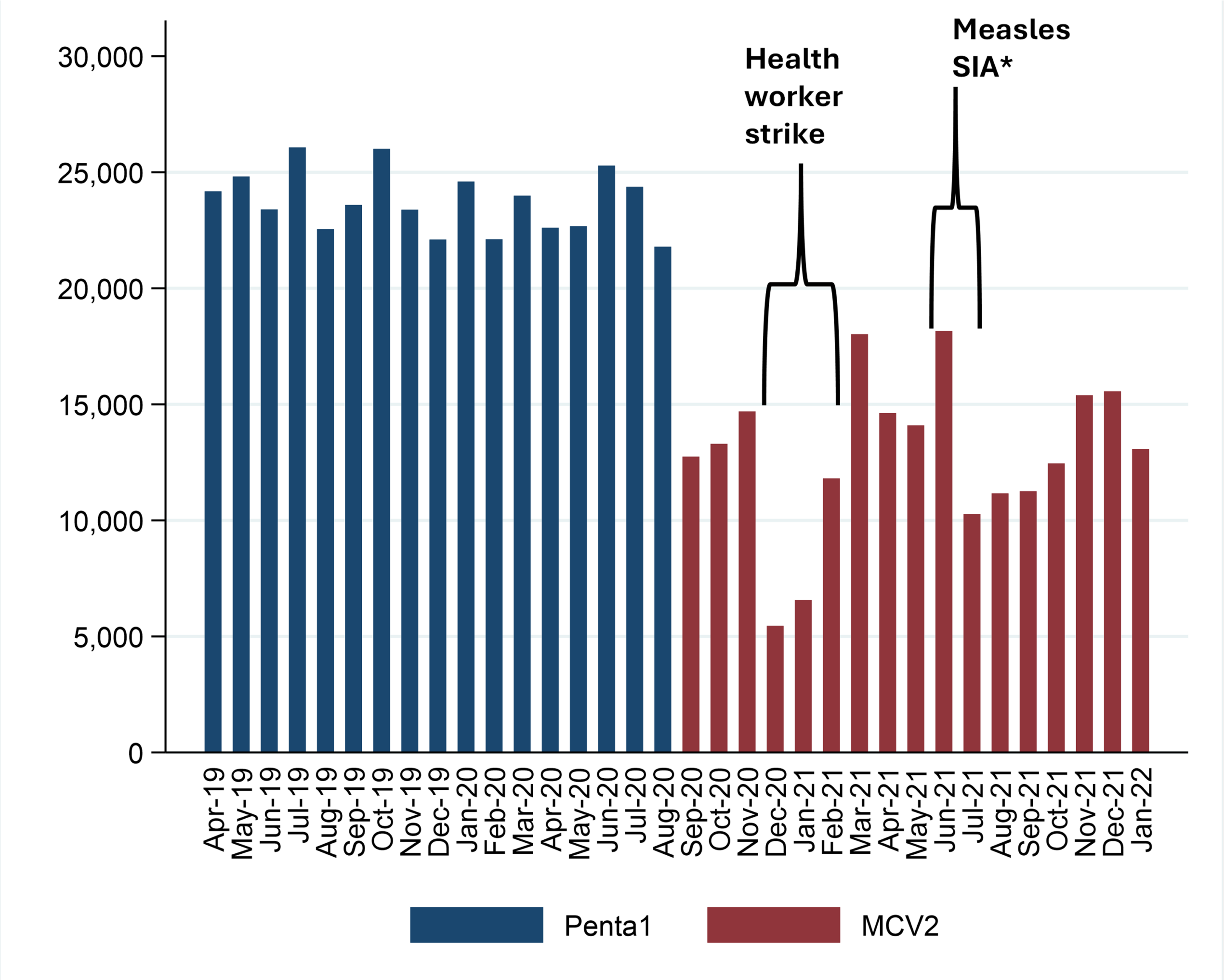

Figure S5: Total vaccines administered monthly across health facilities between April 2019 and January 2022.

*Measles Supplementary Immunisation Activities (SIA) were carried out over 10 days from 25th June to 5th July in 2021, targeting all children aged 9-59 across various counties in Western Kenya.

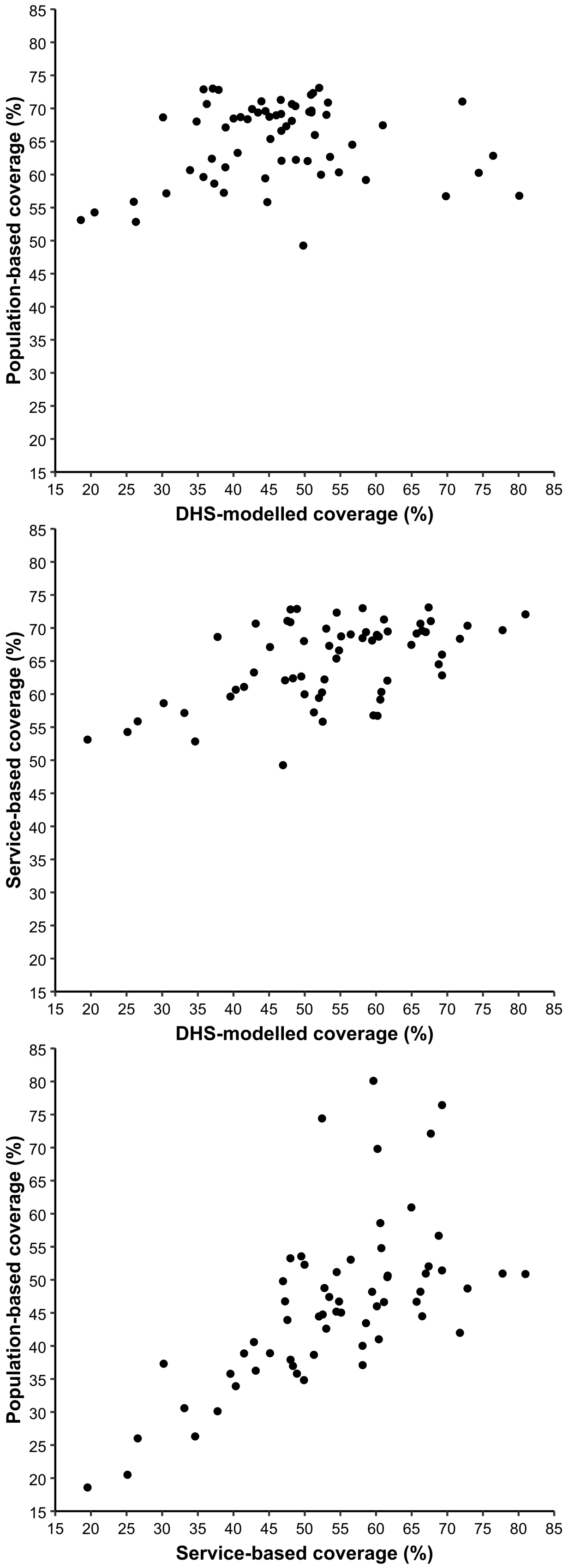

Figure S6: Bivariate plots of MCV 2 coverage estimates at Subcounty level across Western Kenya from population-based against survey-modelled (A), service-based against survey-modelled (B) and population-based against service-based (C) approaches.

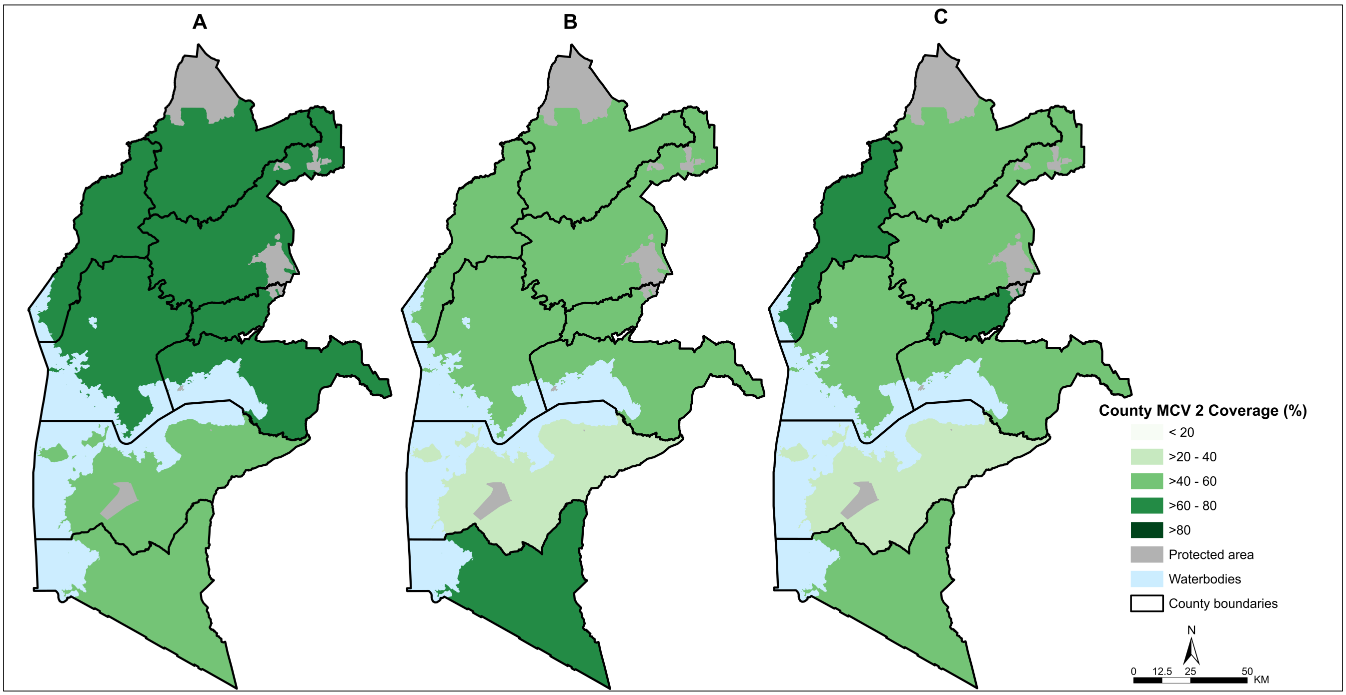

Figure S7: Maps showing the county level MCV 2 coverage estimates across Western Kenya from survey-modelled (A), population (B) and service (C) approaches.

Table S4: Comparison of MCV 2 coverage estimates between survey estimates against the three methods (survey-modelled, Population-based and service-based)

County	Survey estimate (95% CI)	Survey- modelled (%)	Population-based (%)	Service-based (%)
Bungoma	66.9 (55.9 - 76.2)	68.4**	45	52
Busia	84.3 (71.5 - 92)	69.1	54	67
Homa Bay	46.6 (34.9 - 58.6)	56.9**	29	32
Kakamega	81.6 (62.2 - 92.3)	70.8**	44	59
Kisumu	64.7 (51.1 - 76.3)	61.5**	48	53**
Migori	62.3 (51.9 - 71.7)	58.5**	62**	57**
Siaya	54.1 (40 - 67.6)	63.8**	42**	50**
Vihiga	74.8 (60.3-85.3)	68.1**	46	62**

**Estimates within the 95% CI of the survey estimates.
